# Autonomic Dysfunction in Preeclampsia: A Systematic Review

**DOI:** 10.3389/fneur.2019.00816

**Published:** 2019-08-06

**Authors:** Dalia Yousif, Ioannis Bellos, Ana Isabel Penzlin, Mido Max Hijazi, Ben Min-Woo Illigens, Alexandra Pinter, Timo Siepmann

**Affiliations:** ^1^Division of Healthcare Sciences, Center for Clinical Research and Management Education, Dresden International University, Dresden, Germany; ^2^Laboratory of Experimental Surgery and Surgical Research N.S. Christeas, Athens University Medical School, National and Kapodistrian University of Athens, Athens, Greece; ^3^Department of Neurology, Beth Israel Deaconess Medical Center, Harvard Medical School, Boston, MA, United States; ^4^Department of Neurosurgery, University Hospital Carl Gustav Carus, Technische Universität Dresden, Dresden, Germany; ^5^Department of Family Medicine, Semmelweis University, Budapest, Hungary; ^6^Department of Neurology, University Hospital Carl Gustav Carus, Technische Universität Dresden, Dresden, Germany

**Keywords:** preeclampsia, autonomic nervous system modulation, sympathetic activity, parasympathetic activity, heart rate variability, baroreflex sensitivity, muscle sympathetic nerve activity

## Abstract

**Background:** Preeclampsia (PE) is a major obstetric complication that leads to severe maternal and fetal morbidity. Early detection of preeclampsia can reduce the severity of complications and improve clinical outcomes. It is believed that the autonomic nervous system (ANS) is involved in the pathogenesis of PE. We aimed to review the current literature on the prevalence and nature of ANS dysfunction in women with PE and the possible prognostic value of ANS testing in the early detection of PE.

**Methods:** Literature search was performed using Medline (1966–2018), EMBase (1947–2018), Google Scholar (1970–2018), BIOSIS (1926–2018), Web of science (1900–2018); CINAHL (1937–2018); Cochrane Library, Cochrane Database of Systematic Reviews, Cochrane Central Register of Controlled Trials (CENTRAL) and Cochrane Methodology Register (1999–2018). Additionally, the reference lists of articles included were screened.

**Results:** A total of 26 studies were included in the present review presenting data of 1,854 pregnant women. Among these women, 453 were diagnosed with PE, 93.6% (424/453) of which displayed autonomic dysfunction. ANS function was assessed by cardiovascular reflex tests (*n* = 9), heart rate variability (*n* = 11), cardiac baroreflex gain (*n* = 5), muscle sympathetic nerve activity (MSNA) (*n* = 3), and biomarkers of sympathetic activity (*n* = 4). Overall, 21 studies (80.8%) reported at least one of the following abnormalities in ANS function in women diagnosed with PE compared to healthy pregnant control women: reduced parasympathetic activity (*n* = 16/21, 76%), increased sympathetic activity (*n* = 12/20, 60%), or reduced baroreflex gain (*n* = 4/5, 80%). Some of these studies indicated that pressor and orthostatic stress test may be useful in early pregnancy to help estimate the risk of developing PE. However, autonomic function tests seem not to be able to differentiate between mild and severe PE.

**Conclusions:** Current evidence suggests that autonomic dysfunction is highly prevalent in pre-eclamptic women. Among autonomic functions, cardiovascular reflexes appear to be predominantly affected, seen as reduced cardiac parasympathetic activity and elevated cardiac sympathetic activity. The diagnostic value of autonomic testing in the prediction and monitoring of autonomic failure in pre-eclamptic women remains to be determined.

## Introduction

### Rationale

Preeclampsia (PE) is a complex gestational disorder, with a worldwide prevalence of 5–8% (1). Diagnostic criteria for PE have been changing over the years. A new onset hypertension (>140/90 mmHg) after 20 weeks of pregnancy in women who were normally normotensive was recently revised and updated by the American Collage of Obstetricians and Gynecologists (ACOG) to include other complications in case of absence of proteinuria (2).

PE is a major cause of maternal mortality and is regarded as a risk factor for cardiovascular mortality. PE increases the risk of premature death, ischemic and cardiovascular diseases, type 2 diabetes mellitus and hypothyroidism in mothers (3). The complications of PE extend also to the offspring with an increased risk of cardiovascular and metabolic disorders later in life (4). The exact etiology of PE remains elusive, but several theories were proposed. A noteworthy hypothesis postulated that preeclampsia originates from placental dysfunction (5). It seems likely that prohypertensive factors are released into the circulation as a response to diminished adaptive capability of the vasculature in the uteroplacental unit, placental ischemia, and reperfusion (6, 7).

The autonomic nervous system (ANS) has a prominent role in the cardiovascular system adaptation to pregnancy (8). Normal pregnancy is associated with a decrease of parasympathetic and increase of sympathetic activity at rest and upon cardiovascular reflexes stimulation which returnes to baseline after delivery. These changes maintain optimal uteroplacental blood flow (9, 10).

Most studies evaluating the autonomic nervous activity in preeclampsia showed contradicting results. This may be attributed to the fact that some of these studies were cross-sectional or, if longitudinal, compared data in pregnancy with post-partum values, only a few studies were performed before the onset of disease and none were performed before pregnancy. Moreover, most non-invasive methods show large inter-individual variability (11, 12).

### Testing the Autonomic Nervous System

ANS function can be assessed by different tests and techniques. Earlier techniques were limited to some extent by being invasive which limited their routine use, which dictates the development of new, non-invasive techniques with less risk to the mother and fetus allowing incorporation into the routine clinical care in pregnancy.

The most common tests evaluate the cardiovascular reflexes in response to certain maneuvers. Examples are orthostatic stress test, deep breathing test, cold pressor test, Valsalva maneuver, head-up tilt test, isometric hand grip test, and mental stress test. These tests are non-invasive, allowing for bedside evaluation of sympathetic and parasympathetic function by experienced practioners (13).

Heart rate variability (HRV) is a widely used non-invasive clinical tool that provides a valuable measure of parasympathetic function through 24 h monitoring using a Holter device (14). The derived HRV indices are determined in two domains, time domain and frequency domain. The majority of HRV parameters indicate parasympathetic influences (15), while only low frequency (LF) power is influenced by the sympathetic nervous system (16).

Another approach to evaluate the autonomic nervous system activity is to measure the sensitivity of baroreceptors embedded in the carotid sinus and aortic arch walls. Baroreceptor reflex serves as “buffering” mechanism to control sudden fluctuations in blood pressure.

Baroreflex assessment involves simultaneous measurement of heart rate (HR) and blood pressure (BP). Spontaneous fluctuations in BP can be used or BP changes can be provoked by (i) non-invasive procedures (e.g., Valsalva maneuver, lower body negative pressure, or neck suction technique) or (ii) pharmacological agents (e.g., phenylephrine infusion) (12). Both methods rely on the detection of sequences and the regression slope of RR-interval and systolic blood pressure (RRI-SBP) plots yield the baroreflex gain (BRG). Beyond sequence technique, spectral analysis can also be used to evaluate spontaneous corresponding BP-HR changes (Table 1).

**Table 1:** Definition of ANS assessment parameters included in the review.

**Parameter**	**Definition**	**Abnormality**
**Tools and techniques for ANS assessment**		
**1-TESTS OF AUTONOMIC CARDIOVASCULAR REFLEXES**		
**A) Orthostatic stress test**		
Heart rate response to orthostasis	Heart rate response to standing up unaided following a period of lying quietly on a couch. The normal response is an immediate increase in heart rate (around the 15th beat) after standing up followed by a nadir in heart rate (around the 30th beat). The 30:15 ration (of the longest RR interval around the 30th beat to the shortest RR interval around the 15th beat) forms part of the Ewing battery of cardiovascular tests.	30:15 ratio ≤ 1, of baroreceptor origin, indicates the parasympathetic function
Systolic blood pressure response to orthostasis	Systolic blood pressure response to standing up unaided following a period of lying quietly on a couch. The postural drop in systolic blood pressure forms part of the Ewing's battery of cardiovascular tests	A decrease in systolic blood pressure ≥20 mmHg indicates sympathetic dysfunction
**B) Deep breathing test**		
Heart rate variation to deep breathing	Heart rate variation to deep breathing at a rate of 6 breaths per min. The differences between the average of the largest accelerations during inspiration and the largest decelerations during exhalation are calculated. It forms part of the Ewing's battery of cardiovascular tests	HR difference ≤10 characterizes the parasympathetic activity
**C) Cold pressor test**		
Blood pressure response to cold pressor test	Blood pressure response to immersion of hand in a container of cold water for 1–3 min. The diastolic blood pressure response is normally ≥ 15 mmHg.	Diminished responses indicate sympathetic dysfunction and increased responses indicate exaggerated sympathoexcitation
**2- ANALYSIS OF HEART RATE VARIABILITY (HRV)**		
*RMSSD*	The square root of the mean squared differences of successive normal inter-beat (NN) intervals. Time domain estimate of short-term variation of HRV	Reduced values indicate parasympathetic dysfunction
*NN50*	The number of differences in consecutive NN intervals that are longer than 50 ms. Time domain measure.	Reduced values indicate parasympathetic dysfunction
*pNN50%*	NN50 as a percentage of the total number of NN intervals. Time domain measure	Reduced values indicate parasympathetic dysfunction
*SDNN*	The standard deviation of all NN intervals. An estimate of overall HRV. Time domain measure	Reduced values indicate parasympathetic dysfunction
*SDANN*	The standard deviation of the average NN intervals calculated over successive l 5-min segments of the entire recording. Time domain estimate of long-term variation in HRV	Reduced values indicate parasympathetic dysfunction
*SDSD*	The standard deviation of differences between adjacent NN intervals. Time domain measure	Reduced values indicate parasympathetic dysfunction
*HF power*	High-frequency (0.15–0.4 Hz) power of RR interval. Frequency domain measure	Reduced levels indicate reduced parasympathetic activity
*SD 1*	The standard deviation of the Poincare plot (non-linear technique). Short-term HRV parameter	Reduced levels indicate reduced heart rate variability
Respiratory sinus arrhythmia	Rhythmical fluctuations in heart rate periods during inspiration (acceleration) and expiration (deceleration)	Reduced respiratory sinus arrhythmia represents reduced parasympathetic activity
LF power	Low-frequency power of RR interval in the range 0.04–0.15 Hz. Frequency domain measure indicating mainly sympathetic activity (also parasympathetic component). to a smaller extent	Increased levels indicate heightened sympathetic activity
LF/HF ratio	The ratio of low-frequency/high-frequency power of RR intervals. Frequency domain measure of sympatho-parasympathetic balance	Increased levels indicate predominantly heightened sympathetic activity
**3-MICRONEUROGRAPHY**		
Muscle sympathetic nerve activity	Intra-neural recordings of muscle sympathetic nerve activity (MSNA) using tungsten microelectrodes inserted percutaneously into a peripheral nerve (typically peroneal nerve) allow direct measurement of vasoconstrictor sympathetic outflow	Increased levels indicate sympathetic over-activity
**4- BIOMARKERS OF SYMPATHETIC ACTIVITY**		
a) Catecholamines	Catecholamines such as epinephrine, norepinephrine, and their metabolites detected in the plasma or urine (24-h collection) may represent sympathetic activity. Confounding factors include medications, diurnal variations, and concomitant diseases	Increased levels may indicate sympathetic over-activity
b) Plasma neuropeptide Y	Peripheral marker peptide released with norepinephrine following sympathetic activation	Increased levels may indicate sympathetic over-activity
**5- BAROREFLEX SENSITIVITY TESTING TECHNIQUES**		
**a) Sequence technique**		
Regression slope of SBP—RR interval slopes	Blood pressure and RR interval are recorded simultaneously at rest. sequences of 3 or more consecutive beats characterized by a progressive increase or decrease in BP, which results in lengthening or shortening of the RR interval (consecutively) are identified	The reduced slope indicates impaired cardiac cardiac baroreflex gain
**b) Oxford technique**		
Regression slope of SBP and RR interval or heart rate	Phenylephrine (alpha-1 agonist) causes an increase in blood pressure, which results in a baroreflex-mediated slowing of the heart rate	The reduced slope indicates impaired cardiac baroreflex sensitivity
**c) Spectral analysis**		
*a*-index	Spectral analysis of the R-R interval and arterial sytolic blood pressure Computes the gain in the relationship between SAP and RR interval during spontaneous oscillations. The gain in the mid frequency band (0.07–0.14 Hz) between these two signals represents baroreflex gain	Reduced value indicates impaired cardiac baroreflex gain

Another tool that provides an estimate of sympathetic activity is measuring plasma and urinary catecholamines in addition to other blood markers e.g., neuropeptide Y (17). All biomarkers of sympathetic activity share the limitation of being affected by numerous confounding factors that can make interpretation difficult (18).

A recent technique to evaluate sympathetic activity is microneurography during which the sympathetic outflow to the muscle or skin is recorded (19). Muscle sympathetic nerve activity (MSNA) describes well the cardiac sympathetic activity and can be used both for measuring baseline sympathetic activity and response to various stimuli. Its invasiveness and technically challenging nature represent the principal limitations of this method (18).

### Objectives

The objective of this systematic review is to search the existing literature related to the ANS functions in pregnant women diagnosed with preeclampsia, identify the most frequently reported variables and approach their pathophysiological significance. The greater aim is to contribute to forming a basis for the identification of the most useful tools to detect and monitor autonomic dysfunction in pre-eclamptic women.

## Methods

### Study Design

The present systematic review was designed according to the Preferred Reporting Items for Systematic Reviews and Meta-Analyses (PRISMA) guidelines (20). Inclusion criteria were observational studies including pregnant women currently diagnosed with preeclampsia or at increased risk of PE compared to a healthy pregnant control group. Animal studies, studies having no control arm and case report studies were excluded.

### Participants, Interventions, Comparators

Studies in pregnant women diagnosed with preeclampsia of any ethnicity or pregnant women at increased risk of PE due to having a history of PE in a preceding pregnancy were eligible for inclusion.

We excluded studies in women with eclampsia, pre-existing medical disorders like diabetes mellitus, metabolic syndrome, cardiac diseases, renal disease, thyrotoxycosis and chronic hypertension disease, fetal and maternal complications, renal disease, HELLP (hemolysis, elevated liver function, and low platelets) syndrome, diabetes mellitus, hepatic disease, infections, and autoimmune diseases.

Study interventions included techniques used to assess ANS function including such as clinical cardiovascular reflex tests, measurement of plasma and urine level of sympathetic activity biomarkers, heart rate variability testing, baroreflex sensitivity, and microneurography. Comparator group constitutes normotensive pregnant women. Criteria for inclusion were healthy, normotensive women with appropriately grown fetuses, normal blood pressure throughout pregnancy (BP < 140/90 mm Hg), gave birth to healthy children, uncomplicated pregnancy, matched at gestational age to the PE group. Pregnant women having histories of hypertension, diabetes, cardiovascular or renal disease before pregnancy; taking antihypertensive medication or any medications other than iron supplementation were excluded.

### Systematic Review Protocol

**Figure 1:**
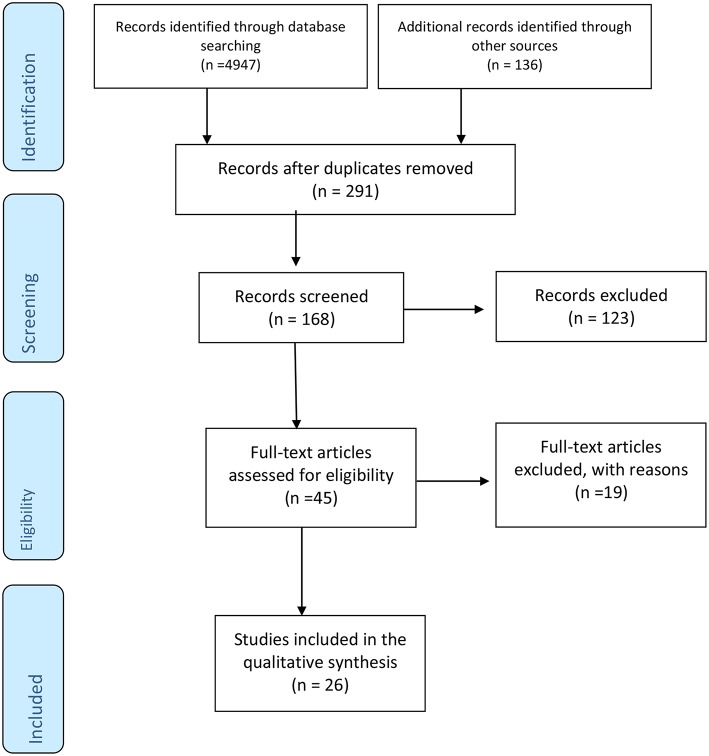
PRISMA diagram showing systematic search process, which started with 4,947 articles identified through searching Medline, EMBase, BIOSIS, Web of Science, CINAHL, and the entire Cochrane Library in addition to 136 articles retrieved from the references list of the selected studies. Duplicate articles were excluded. From 168 articles screened, 123 articles were excluded because they didn't meet the inclusion criteria (animal studies, letter to the editor, case reports). The number of full articles assessed for eligibility was 45, of which 19 articles were found not- eligible due to lacking a PE group, lacking a healthy control group or including non pregnant women with history of PE. Finally, 26 studies were found eligible for inclusion in our review.

Literature search was performed by using Medline (1966–2018), Web of science (1900–2018); CINAHL (1937–2018), EMBase (1947–2018); Google Scholar (1970–2018), BIOSIS (1926–2018); the Cochrane Library, Cochrane Database of Systematic Reviews, Cochrane Central Register of Controlled Trials (CENTRAL), and Cochrane Methodology Register (1999–2018). Additionally, the reference lists of the selected studies were also screened. No restrictions on language or date were applied during the literature research. The date of the last search was set on 1 August 2018. The search strategy included the search term “preeclampsia” in combination with each of the following terms; “autonomic,” “sympathetic,” “parasympathetic,” “vagal,” “heart rate variability,” “baroreflex,” “catecholamine,” “epinephrine,” “norepinephrine,” “adrenaline,” “noradrenaline,” “Valsalva,” “hand grip,” “cold pressor,” “orthostasis,” and “baroreceptor gain” and is schematically presented in the PRISMA flowchart (Figure 1).

Two authors (Yousif D and Bellos I) independently screened all articles for eligibility, potential disagreements were resolved by the consensus among all authors.

### Data Sources, Studies Selection, and Data Extraction

The studies were selected in three consecutive steps. First, the titles and abstracts of all electronic articles were screened to assess their eligibility according to the inclusion criteria. Second, the selected articles were retrieved as full texts. In the third stage, all observational studies that evaluated autonomic functionality in women with preeclampsia and healthy normotensive pregnant women were included. Animal studies, case reports, review articles as well as conference abstracts were excluded. Two reviewers “Yousif D and Bellos I” independently extracted data from the appropriate trials using a pre-designed standard form. The retrieved data comprised: author names, year of publication, study design, exclusion criteria, number of patients, maternal age, gestational age, parity, gravidity, type of autonomic function assessment, catecholamine plasma concentration (adrenaline, noradrenaline, dopamine), neuropeptide Y level, time and frequency indices for heart rate variability, heart rate, and blood pressure variability in response to cardiovascular reflex tests (30:15 ratio, Valsalva ratio), MSNA and baroreceptor sensitivity index.

### Data Analysis

Findings from the eligible studies were aggregated to produce a qualitative summary structured around the study design, sample size, type and outcome of intervention and population characteristics.

## Results

### Study Selection and Characteristics

Out of 45 eligible full-text articles, 26 observational studies were finally included in the present review, 22 were cross-sectional studies and 4 were longitudinal studies.

Nineteen studies were excluded after reading the full text based on various reasons: 13 studies included other types of pregnancy-induced hypertension but not preeclampsia. Two studies did not include a control arm of normotensive healthy pregnant women. Four studies included women who were formerly diagnosed with preeclampsia but not pregnant during the study.

One thousand eight hundred fifty-four was the total number of women included. Among them, 453 subjects were diagnosed with PE, 1,104 subjects were healthy pregnant controls, 150 subjects were included as a normotensive non-pregnant group, and 147 subjects represented other hypertensive pregnancy disorders (chronic hypertensive pregnancy, pregnancy-induced hypertension PIH, and gestational hypertension) in 8 studies.

The methodological characteristics (study design, exclusion criteria, examined test, gestational age, maternal age) and NOS (new castle ottawa scale) scores are described in Table 2.

**Table 2:** Characteristics of studies included in the review.

**References**	**Study design**	**Exclusion criteria**	**Examined test**	**Maternal age (years)**		**Gestational age (weeks)**		**NOS score**
				**PE**	**Control**	**PE**	**Control**	
**BIOMARKERS**								
Egerman et al. (21)	Case-control	Chronic hypertension	Serum neuropeptide Y	23.7 ± 1.0	22.1 ± 1.1	35.7 ± 1.4	36.9 ± 1.0	9
Manyonda et al. (22)	Case-control	Multigravidity	Cord blood noradrenaline	28.5 (21–34)	28.4 (19–39)	31.6 (28–37)	36 (28–42.7)^*^	8
Øian et al. (23)	Case-control	History of hypertension, chronic kidney disease	Arterial/venous epinephrine, norepinephrine, dopamine	28 ± 2	27 ± 1	32.5 ± 1.3	33.5 ± 1.3	8
Beilin et al. (24)	Prospective	NR	Plasma renin activity, angiotensin II, norepinephrine, epinephrine	NR		NR		7
**CARDIOVASCULAR REFLEX TESTS**								
Chaswal et al. (25)	Case-control	NR	Heart rate variability, deep breathing test, orthostatic stress test	26.88 ± 3.52	26.35 ± 2.53	NR		8
Lakhno (26)	Case-control	Multiple pregnancies, eclampsia, history of hypertension diabetes mellitus, any cardiac/renal disease, thyrotoxicosis	24-h Holter heart rate variability	25.6 ± 6.8	26.5 ± 4.1	36.8 ± 2.2	37.1 ± 3.6	8
Musa et al. (27)	Case-control	History of hypertension, diabetes mellitus, renal disease, liver/thyroid disease	Heart rate variability	30.6 ± 6	30 ± 6.2	33.8 ± 4.3	32.9 ± 4	8
Flood et al. (28)	Prospective	Pregnancy loss/delivery before 28 weeks	Heart rate variability	27.9 ± 6.3	26.4 ± 5.1	28		6
Yokuşoglu et al. (29)	Case-control	Multiple pregnancies, intrauterine growth restriction, HELLP syndrome	24-h Holter heart rate variability	29 ± 4	27 ± 4	33 ± 3	39 ± 6^*^	9
Swansburg et al. (30)	Case-control	Age < 16 years, multiple pregnancies, premature rupture of membranes, Hirschsprung's disease	Heart rate variability, orthostatic stress test, fetal heart rate, spontaneous Baroreflex sensitivity	28.3 ± 6.6	29.9 ± 4.7	37 ± 2.6	35.8 ± 2	7
Rang et al. (31)	Prospective	Intrauterine growth restriction without hypertension	Orthostatic stress test, paced breathing test	28.6 ± 2.3	29.9 ± 4	Pre-pregnancy, 6, 8, 12, 16, 20, 32, 12 postpartum		8
Miyake et al. (32)	Case-control	Mild preeclampsia	Orthostatic stress test	29.9 ± 3.4	29.9 ± 4.3	34.6 ± 3.6	36.1 ± 2.2	7
Woisetschläger et al. (33)	Prospective	History of hypertension, current antihypertensive treatment, fever, diabetes mellitus	Cold pressor test	28 ± 6	27 ± 5	17.3 ± 1.8	18.5 ± 2.2	9
Yang et al. (34)	Case-control	Diabetic neuropathy, any cardiac disease, any drug intake	Heart rate variability	30 ± 1	28 ± 1	35 ± 1	34 ± 1	8
Lewinsky and Riskin-Mashiah (35)	Case-control	Diabetes mellitus, any drug intake except iron supplementation	Heart rate variability, supine pressor test	24 ± 5	25 ± 4	35 ± 4	33 ± 3	6
Eneroth and Storck (36)	Case-control	History of hypertension, diabetes mellitus, renal disease, any drug intake	Heart rate variability	NR		33.4 ± 1.6	33.0 ± 2	8
Ahmad et al. (37)	Cross-sectional	Recent history of diarrhea and vomiting, hematocrit <32%	Orthostatic stress test	24.8 ± 2	*1st trimester:* 26.4 ± 3.16 *2nd trimester:* 29 ± 2.3 *3rd trimester:* 28. ± 2.4	32 ± 1.9	*1st trimester:* 9.8 ± 3.16 *2nd trimester:* 20.7 ± 1.68 *3rd trimester:* 33.5 ± 2.35	8
Airaksinen et al. (38)	Case-control	Any cardiovascular or renal disease, diabetes mellitus	Deep breathing test, orthostatic stress	28 (17–37)	28 (23–38)	35 (32–39)	34 (32–38)	7
**MUSCLE SYMPATHETIC NERVE ACTIVITY**								
Fischer et al. (39)	Prospective	History of hypertension, cardiac/renal disease	Muscle sympathetic nerve activity, forearm blood flow, blood pressure after forearm occlusion	31.7 ± 3.9		22 ± 4, 33 ± 5, and 26 ± 6 postpartum		8
Greenwood et al. (40)	Case-control	Secondary hypertension, diabetes mellitus, malignancy, neurologic dysfunction	Muscle sympathetic nerve activity	27.5 ± 1.5	28 ± 1.2	35 ± 1.1	35 ± 0.6	8
Schobel et al. (41)	Case-control	History of hypertension, cardiac/renal disease	Muscle sympathetic nerve activity	26 ± 1	26 ± 1	33 ± 1	32 ± 1	7
**BAROREFLEX SENSITIVITY**								
Weber et al. (42)	Case-control	Diabetes mellitus, cardiac/renal disease, multiple pregnancy	Heart rate variability, baroreflex sensitivity	30.3 ± 6.3	31.9 ± 5.0	33 ± 3	33 ± 3	9
Faber et al. (43)	Cross-sectional	NR	Heart rate and blood pressure variability, baroreflex sensitivity	27 (22–31)	28 (24–31)	32 (30–36)	35 (32–37)	8
Silver et al. (44)	Case-control	History of hypertension, diabetes mellitus, multiple pregnancy, vasoactive medication, or intravenous hydration	Vagal baroreflex gain	25.4 ± 4.5	25.2 ± 4.7	34.1 ± 2.9	34.0 ± 3.5	9
Molino et al. (45)	Case-control	History of hypertension, cardiac/renal disease	Baroreflex gain, interbeat interval	32 (29–33)	31 (30–34)	35.0 (32.0–36.0)	32.5 (28.5–36.5)	7
Seligman (46)	Case-control	NR	Baroreflex sensitivity-phenylephrine or angiotensin II infusion	NR				3

*The table shows the study design, the exclusion criteria, the examined ANS test, maternal age (years), gestational age (weeks), and the risk of bias scores by Newcastle Ottawa score (NOS) for each study. Data that was not reported in the studies were denoted NR*.

* *:statistically significant difference between the two groups*.

The definition of Preeclampsia was inconsistent between studies. In 10 studies, PE was defined according to the International Society for the Study of Hypertension in Pregnancy which was “evidence of elevated blood pressure (evidence of antihypertensive drug treatment and/or evidence of systolic blood pressure 140 mmHg and/or diastolic blood pressure 90 mmHg during pregnancy on two or more occasions) and detection of proteinuria defined as 0.3 g/day or greater in a 24-h specimen or 0.3 g/l (1. dipstick) or greater in a random urine determination” (47).

In three studies, PE was defined according to the recommendations of National High Blood Pressure Education Program Working as “proteinuria > 300 mg per 24 h, no history of hypertension, cardiovascular, or renal disease, and blood pressure values exceeding 140/90 mmHg after the 20th week of gestation, confirmed by two consecutive readings, with blood pressure reverting to normal within 2 months after delivery” (48).

In three other studies, PE was defined according to the clinical criteria established by The American College of Obstetricians and Gynecologist as the “occurrence of hypertension defined as systolic blood presseure ≥ 140 mm Hg or diastolic blood pressure ≥ 90 mm Hg after 20 weeks of gestation in woman who is normotensive before, and proteinuria defined as presence of 300 mg or more of protein in 24 h urine sample or > 2+ on dipstick” (49).

Diagnostic criteria for PE was not reported in two studies (24, 46).

Three studies have included severe PE patients diagnosed according to different criteria. In one study, severe PE was defined according to a diastolic blood pressure of more than 110 mmHg (27). While in another study, severe PE was defined as “when two or more of the following findings evolved after 24 weeks of gestation, systolic blood pressure of at least 160 mmHg or diastolic blood pressure at least 110 mmHg on two or more occasions, separated at least a day and measured while the patient was on bed-rest, proteinuria of at least 5 g/24 h and subjective symptoms of headache, dizziness, visual disturbances reported by the mother” (38).

A third study diagnosed severe PE as having “blood pressure higher than 160 mmHg systolic and 110 mmHg diastolic or (and) thrombocytopenia, serum creatinine more than 1.1 mg/L, elevated blood concentration of liver transaminases to twice normal concentration, pulmonary edema, cerebral, or visual disturbances” (26).

Exclusion criteria for PE patients in 58% of the studies were a history of chronic hypertension, diabetes mellitus, any cardiac/renal disease, liver/thyroid disease, and current antihypertensive treatment. Multiple pregnancies were exclusion criteria in 23% of studies. Other exclusion criteria in 19 % of the studies were: pregnancy loss (delivery before 28 weeks), intrauterine growth retardation, HELLP syndrome, age < 16 years, premature rupture of membranes, Hirschsprung's disease, diabetic neuropathy, a recent history of diarrhea and vomiting and hematocrit < 32%.

It is important to note that in 88.5% of the studies, the maternal age was comparable between the PE group and the healthy pregnant control group and in 77% of the studies, the PE group was matched for gestational age with a healthy pregnant control group. The outcomes of each study included in the review are presented in Table 3.

**Table 3:** List of autonomic measures for studies included in the review.

**References**	***n* (PE/control)**	**Autonomic measure**		
			**PE**	**Control**
**BIOMARKERS**				
Egerman et al. (21)	12/12	NPY (ng/mL)	33.3 ± 3.6	32.2 ± 3.5
Manyonda et al. (22)	12/26	Venous NE (ng/ml)	1.93 (0.20)^*^	1.15 (0.12)
		Venous EPI (ng/ml)	0.25 (0.035)^*^	0.23 (0.033)
		Cord NE venous plasma (ng/ml)	1.94 (0.26)^*^	1.16 (0.09)
		Cord NE arterial (ng/ml)	2.95 (0.97)^*^	1.8 (0.18)
Øian et al. (23)	13/13	Arterial EPI (ng/ml)	125 (24)^*^	43 (5)
		Arterial EPI (ng/ml)	337 (39)^*^	243 (19)
		Arterial dopamine (ng/ml)	214 (77)^*^	32 (6)
		venous NE (ng/ml)	67 (10)^*^	37 (6)
		venous NE (ng/ml)	299 (38)	256 (24)
		venous dopamine (ng/ml)	73 (11)^*^	41 (7)
Beilin et al. (24)	8/10	Plasma renin activity (ng/ml/h)	2.22^*^	7.55 (10)
		Plasma angiotensin 2	28.36^*^	57.9
		Free plasma EPI	0.028^*^	0.024
		Free plasma NE	0.308	0.229
**CARDIOVASCULAR REFLEX TESTS**				
Chaswal et al. (25)	40/40	30:15 (OS)	1.13 (0.11)	1.22 (0.11)^*^
		HR (DB) (bpm)	13.48 (6.12)	22.6 (8.18)^*^
		SDNN, ms	26.17 (2.7)	34.98 (1.1)^*^
		RMSSD, ms	18.04 (2.33)	34.68 (2.62)^*^
		LF, ms^2^	125.56 (19.36)	192.9 (19.7)^*^
		LF/HF	2.9 (2.4)^*^	1.7 (1.5)
		HF, ms^2^	132.28 (37.8)	447.24 (63)^*^
Lakhno (26)	76/30	SDNN, ms	92.99 (10)^*^	111.8 (14.1)
		RMSSD, ms	19.5 (5.5)^*^	41.6 (8.5)
		LF, ms^2^	271 (51.6)^*^	349.5 ± 42.6
		HF, ms^2^	90.85 (17.5)^*^	375.4 ± 56.1
		LF/HF	3.35 (0.85)^*^	0.9 ± 0.3
Musa et al. (27)	60/60	LF Norm, ms^2^	49.80 (16.25)^*^	44.55 (19.15)
		Ln LF/HF	0.04 (0.68)^*^	−0.28 (0.91)
		HF norm, ms^2^	45.08 (15.29)^*^	55.87 (19.56)
		Ln LF	4.01 (1.58)^*^	3.49 (1.23)
Flood et al. (28)	27/332	HF-HRV, geometric mean	363 (197, 668)	358 (314, 408)
		LF-BPV (SBP), geometric mean (95% CI)	8.9 (7.0, 11.3)	9.7 (9.1, 10.3)
		LF-BPV (DBP), geometric mean (95% CI)	4.3 (3.5, 5.3)	4.1 (3.9, 4.4)
Yokuşoglu et al. (29)	34/29	SDNN, ms	109 ± 52^*^	130 ± 56
		SDANN, ms	80 ± 33^*^	108 ± 37
		HRV-triangular index	27 ± 9^*^	32 ± 10
Swansburg et al. (30)	9/18	PNS(HF/TP)	0.22 (0.15)^*^	0.11 (0.14)
		SNS(HF/LF) lying to standing	1.6–4.5^*^	1.9–2.8
Rang et al. (31)	8/30	Phase difference in the supine position LF	8 week = 77 (18)^*^	64 (15)
			12 week = 77 (22)^*^	61 (19)
			20 week = 79 (37)^*^	63 (30)
		OS- Delta MAP-, mmhg	16 week = 26 (18–33)^*^	15 (10–17)
			20 week = 30 (24–37)^*^	12 (10–20)
Miyake et al. (32)	17/138	TPR (%) postural change	45 (4)^*^	18 (10)
		SBP (%) postural change	−7.5 (0.8)^*^	−5 (0.5)
		HF (%) postural change	−50 (19)^*^	48 (20)
		LF/HF (%) postural change	390 (210)^*^	80 (20)
Woisetschläger et al. (33)	10/113	CP-SBP, mmHg	14.2 (5.5)^*^	8.5 (7.2)
		CP-DBP, mmHg	7.3 (4.9)^*^	3.9 (4.7)
Yang et al. (34)	17/17	Ln (LF/HF)	1 (1.3)^*^	0.3 (0.5)
		LF% (nu)	60 (65)^*^	55 (60)
		Ln HF, ms^2^	3.57 (0.4)	5.79 (0.22)^*^
Lewinsky and Riskin-Mashiah (35)	15/25	VLF, s^2^/Hz	288 (214)/556 (322)^*^	281 (225)/278 (194)
		HF, s^2^/Hz	78 (79)/78 (78)	52 (52)/49(59)
		TP, s^2^/Hz	544 (322)/878 (397)^*^	472 (341)/475 (291)
Eneroth and Storck (36)	15/15	Average R-R interval (24h), ms	770 ± 133^*^	690 ± 50
		LF power (24 h), ms	925 ± 362	839 ± 288
		HF power (24 h)	597 ± 742	655 ± 337
		Average R-R interval, ms (daytime)	736 ± 132^*^	642 ± 47
		Average R-R interval, ms (night time)	824 ± 159	789 ± 77
Ahmad et al. (37)	•16/78 •1st trimester = 25	Rate of HR change (bpm)	0.83 (0.16)^*^	1st = 0.94 (0.13)
	•2nd trimester = 25			2nd = 0.9 (0.14)
	•3rd trimester = 28			3rd = 0.72 (0.13)^*^
		Lying BP, mmHg	135/90 ± 13/6	3rd:105/64 ± 15/11
		Standing BP, mmHg	146/100 ± 17/14	111/70 ± 12/6
		Delta HR, bpm	12 (3)^*^	3rd = 16 (3)
Airaksinen et al. (38)	14/11	OS-30:15 ratio	1.15 (0.17)^*^	1.39 (0.14)
		DB-HR, bpm	12 (4)^*^	18 (6)
		SBP standing up, mmHg	−11 (32)	4 (12)
Fischer et al. (39)	6/16	MSNA (burst/min)	M1: 21 (9)^*^, M2: 29 (14)^*^ vs. postpartum M3: 9 (5)	
		Gestational MSNA, burst/min	M1: 21 (5), M2: 27 (6), M3: 7 (4)	M1: 21 (11), M2: 30 (16), M3: 9 (6)
Greenwood et al. (40)	11/11	s-MSNA (impulses/100 beats)	PE = 62 (10.8)^*^	39 (7.7), PIH: 128 (23.4)^*^
		MSNA (bursts/100 beats)	PE = 51 (7.1)	28 (2.3)^*^, PIH:62 (3.8)^*^
Schobel et al. (41)	9/8	SNA, bursts per min	33 (3)^*^	10 (1)
		MSNA, burst/min	6 PE –During preg: 36 (4)^*^ vs. after delivery: 13 (2)^*^	
Weber et al. (42)	24/72 Early onset: 10/30 Late onset: 14/42	SDNN, ms	46.5 ± 17.4^*^ late 33.2 ± 10.7 early 41.0 ± 16.1 all	37.1 ± 12.2 late 35.2 ± 9.9 early 36.5 ± 11.2 all
		RMSSD, ms	33.3 ± 18.9^*^ late	21.0 ± 8.9 late
			17.7 ± 9.5 early	19.9 ± 10.3 early
			26.8 ± 17.3^*^all	20.5 ± 9.4 all
		HF, ms^2^	5.58 ± 0.98 ^*^late	4.87 ± 0.93 late
			4.44 ± 1.18 early	4.81 ± 0.96 early
			5.11 ± 1.19 all	4.84 ± 0.94 all
		BRS, ms/mmHg	13.6 ± 7.0^*^ late	10.4 ± 3.8 late
			9.1 ± 4.4 early	10.3 ± 4.9 early
			11.7 ± 6.4 all	10.4 ± 4.2 all
Faber et al. (43)	44/80	BPV: SDNN, ms	9 (8–10)^*^	8 (7–9)
		BPV: RMSSD, ms	2.5 (2.1–2.8)^*^	3.1 (2.8–3.6)
		brady_2.5-5, NU	16 (8–25)^*^	11 (6–18)
		tachy_slope, ms/mmHg	6.9 (5.7–9.6)	6.4 (4.9–9.3)
		HRV: SDNN, ms	43 (29–51)	44 (31–53)
		HRV: RMSSD, ms	18 (12–28)	16 (10–24)
		HRV: LF, ms^2^	0.15 (0.10–0.20)	0.16 (0.07–0.25)
Silver et al. (44)	20/20	Baroreflex gain: VM- (ms/mmHg)	6.6 ± 2.5^*^	10.1 ± 3.2
		DB-BP (ms/mm Hg)	10.0 ± 5.9	14.1 ± 6.9
		Spontaneous HRV (ms/mmHg)	7.2 ± 2.6^*^	10.8 ± 4.1
Molino et al. (45)	9/8	BRG index (a-index) ms/mmHg	At rest: 5.60 (5.25–6.90)^*^	At rest: 7.98 (6.71–9.93)^*^
			Standing: 4.07 (3.70–6.92)	Standing: 5.70 (5.24–7.18)
		IBI variability		
		LF (NU)	At rest: 0.62 (0.47–0.69)	At rest: 0.48 (0.35–0.76)
		HF (NU)	Standing: 0.56 (0.48–0.66)	Standing: 0.62 (0.47–0.76)
Seligman (46)		BRS, ms/mmHg	3.6^*^	10.3 (19–9)

*Data extracted include author name, the sample size for PE group and healthy pregnant control group and the results of ANS assessments performed in each study*.

Values denoted by

* represent statistical significance. 30:15, ratio of the longest inter-beat (RR) interval around the 30th beat to the shortest RR interval around the 15th beat; BP, blood pressure; BPV, blood pressure variability; brady_2.5-5, Number of brady cardiac baroreflex fluctuations with a slope < 50 ms/mmHg; BRG, baroreflex gain (ms/mmHg); BRS, baroreceptor sensitivity; CP, cold pressor test; DB, deep breathing test; DBP, diastolic blood pressure; EPI, epinephrine; HF, high-frequency power in the range 0.15–0.40 Hz; HR, heart rate; HRV, heart rate variability; HRV-triangular index, integral of the density of the RR interval histogram divided by its height; IBI, interbeat interval; LF, low-frequency power in the range 0.04–0.15 Hz; LF/HF ratio, low frequency to high-frequency ratio; MAP, mean arterial pressure; MSNA, Muscle sympathetic nerve activity; NE, norepinephrine; NN, inter-beat interval; NPY, neuropeptide Y; NU, normalized units; OS, orthostatic stress test; PNS (HF/TP), parasympathetic indicator; RMSSD, square root of the mean of the sum of the squares of difference between adjacent NN intervals; RR interval, the time between two R-peak of ECG heart-beat waveform; SBP, systolic blood pressure; SDANN, standard deviation of the averages of NN intervals in all 5-min segments of the entire recording; SDNN, standard deviation of all NN intervals; SNA, sympathetic nerve activity; SNS, sympathetic indicator; tachy_slope, slope of the regression line between all tachycardiac baroreflex fluctuations; TPR, total peripheral resistance; TP, total power; VLF, very low frequency; VM, Valsalva's maneuver.*:statistically significant difference.

### Synthesized Findings

Each study was classified according to the type of autonomic test performed and the normality of the PE group response in comparison to the control group into either normal or abnormal.

A total of 33 autonomic tests was performed in the included studies. Most of the studies (78%) used one ANS assessment test, 19% of the studies used two assessment tests (30, 31, 38, 42, 43), and one study used three types of tests (25).

Table 4 summarizes the number and the outcome for each ANS test.

**Table 4:** Summary of the outcome of autonomic assessment.

**Autonomic test**	**Abnormal response**	**Normal (no significant difference between PE and control)**	**Number of tests**
Heart rate variability	8	3	11
Orthostatic stress	6	0	6
Deep breathing	3	0	3
Cold pressor	1	0	1
Baroreceptor reflex gain	4	1	5
Biomarkers of sympathetic activity	2	2	4
Muscle sympathetic nerve activity	2	1	3
Total number of tests	26	7	33

*The table shows types and number of ANS tests performed within 26 studies included in the review; Heart rate variability HRV = 11, Orthostatic stress = 6, deep breathing test = 3, cold pressor test = 1, Baroreflex sensitivity = 5, Biomarkers of sympathetic activity' = 4, Muscle sympathetic nerve activity = 3. A total number of tests performed was 33*.

*The number of studies with abnormal results for each test type is described, HRV tests showed (72%), abnormal response cardiovascular reflex testing (orthostatic stress, deep breathing, and cold pressor test) results showed a 100%abnormal response. Baroreflex sensitivity showed an 80% abnormal response, Biomarkers of sympathetic activity showed 50 % abnormal response and muscle sympathetic nerve activity showed 66% abnormal; response. The overall response from all tests performed showed 78.8% abnormal ANS response with cardiovascular reflex testing providing the most consistent results*.

Heart rate variability was analyzed in 11 studies, either alone in six studies or in combination with one or two tests in five studies. Both time and frequency domains were computed in seven studies (25–27, 30, 36, 42, 43), while in one study only time domain parameters were shown (29). Three studies reported only frequency domain indices (28, 34, 35).

HRV tests showed significant ANS dysfunction presented as elevated sympathetic activity and suppressed parasympathetic activity in eight studies, while three studies showed no significant difference between the PE group and the control group (28, 36, 43).

One prospective cohort study evaluated the predictive value of spectral analysis of heart rate and blood pressure for hypertensive diseases of pregnancy at 28 weeks of pregnancy. Although useful for pregnancy-induced hypertension; it was not able to detect women who developed PE afterward in pregnancy (28).

Two studies had further categorized PE patients into mild and severe based on diastolic blood pressure of <110, or >110 mmHg, respectively, the results of which were contradicting. One study did not show any significant difference comparing time and frequency domain measurements between mild and severe PE (27). On the other hand, a study by Lakhno (26) showed that mean sympathovagal balance (LF/HF) increased gradually in association with the progredient severity of PE even when subjects were on antihypertensive medications.

Additionally, they investigated correlations between the maternal and fetal time domain and noticed a loss of fetal-maternal hemodynamic coupling in case of severe PE vs. a positive weak correlation in case of mild PE (26).

Orthostatic stress test, cold pressor test, and deep breathing test represented the cardiovascular reflex test procedures performed in nine studies.

Orthostatic stress test was performed in six studies, the results showed consistently sympathetic dominance and parasympathetic withdrawal in PE patients compared to the normotensive control group (25, 30–32, 37, 38, 50).

One prospective study aimed to evaluate the value of blood pressure response to orthostatic challenge for early prediction of women who develop preeclampsia in the second half of pregnancy. Responses to orthostatic stress were recorded at 8-time points, before pregnancy, at first, second, and third trimester and 15 weeks after delivery. Results showed that, for women who developed PE later in pregnancy, significantly higher blood pressure drop to orthostatic stress before pregnancy, during the first, and the second trimester when compared to women with uncomplicated pregnancy. This results support the hypothesis that sympathetic hyperactivity develops early in pregnancy before the clinical presentation of PE and may play a role in the (31).

Cold pressor test was performed in one prospective cohort study, as one of the cardiovascular reflex test procedure, early in pregnancy between 16 and 20 gestational weeks (33). Results showed a significant increase in sympathetic activity in subjects who developed PE later in pregnancy in comparison to subjects with uncomplicated pregnancy which may suggest its value as an early detection tool for the risk of PE. This can be explained by an increased vasoconstrictive response to a physiological stimulus in women with preeclampsia as a sign of increased vascular reactivity before clinical manifestation of the disease.

Deep breathing test was performed in three studies, all showed reduced parasympathetic activity in the PE group (25, 38, 51).

It is worth mentioning that the results of the studies that showed abnormal ANS function in PE patients were consistent regarding the pattern of the ANS dysfunction, which was inhibition of the parasympathetic tone and/or increased sympathetic activity, except for one study which reported that PE patients exhibited a significant increase in the time domain parameters of heart rate variability and baroreflex sensitivity compared to the control group (42). This controversy could be explained by subject selection. Weber et al had further classified patients in the PE group according to the onset of diagnosis into early-onset preeclampsia (PE diagnosed at < 34+0 weeks of gestation) and late-onset preeclampsia (PE diagnosed at ≥ 34+0 weeks of gestation). Amelioration of autonomic function was observed only in patients with late-onset PE.

Biomarkers of sympathetic activity were used to assess ANS in four studies. Results from these studies were controversial. In one of them, neuropeptide Y plasma level was determined and no significant difference was found between the PE group and healthy control group (21). Three studies measured catecholamine (adrenaline, noradrenaline) blood levels. Two studies reported significantly increased catecholamine levels in the PE group when compared to the healthy control group (22, 23).

Kjeldsen et al. further compared arterial and venous catecholamine levels. His results showed that both arterial and venous levels of adrenaline and dopamine were significantly elevated where only arterial but not venous noradrenaline levels were significantly increased in the PE group (23). A small study showed no significant difference in the biomarkers of ANS activity in the PE group in comparison to the healthy pregnant control group (24). Beilin et al. evaluated the diurnal pattern of catecholamine (adrenaline and noradrenaline) and pressor hormones (renin and angiotensin 2) in normal and hypertensive pregnancies. Results of this study showed that levels from the 2 pressor hormones fell progressively in all groups and were lower in PE group, the diurnal pattern of noradrenaline with lower levels at midnight was not observed in PE while plasma adrenaline level showed no significant difference between PE and healthy control group.

MSNA was measured in three studies, two of which showed sympathetic hyperactivity in PE compared to healthy pregnant controls (40, 52). A small prospective longitudinal study evaluated whether MSNA can predict women who develop PE later in pregnancy. In this study MSNA was recorded at three-time points (twice during pregnancy and one time postpartum) in healthy pregnant women at increased risk of developing PE (39). Results from Fischer et al showed significant increased MSNA during normal pregnancy compared to postpartum values but did not show a significant difference between MSNA values in women who developed PE later in pregnancy and women who had a normal pregnancy. This showed that MSNA has no value in predicting the risk of PE in high-risk patients.

Cardiac baroreflex gain was assessed in five studies. Three studies showed that PE women exhibit significant reduction in baroreflex gain (44–46).

Techniques used for determination of BRG were variable. Silver et al. and Molino et al. used cross-spectral analysis of parallel spontaneous heart rate and blood pressure changes to measure BRG. BRG was computed as alpha index, i.e., “the square root of the ratio of the powers of interbeat interval in the low-frequency range to corresponding spectral components of systolic blood pressure” (44, 45).

Seligman used an invasive technique, which is using intravenous phenylephrine or angiotensin to induce bradycardia. The sensitivity of baroreflex was calculated in milliseconds of cardiac slowing per millimeter rise in systolic pressure (46).

Results from one large cross-sectional study comparing heart rate variability, blood pressure variability and baroreflex gain across different pregnancy-induced hypertensive disorders (chronic hypertension and pregnancy-induced hypertension) showed that although a significant elevation in blood pressure variability was seen in the PE group, this increase did not lead to elevated spontaneous baroreflex (43). In this study, the sequence method was used to estimate BRG. This method is based on identifying consecutive cardiac beats in which an increase in systolic blood pressure is accompanied by an increase in heart rate, or in which a decrease in systolic blood pressure is accompanied by a decrease in heart rate. The regression line between the systolic blood pressure and heart rate produces an estimate of BRG. Similarly, the sequence technique was used by Weber et al. however, results obtained were contradicting. Baroreflex gain was significantly increased only in women with late-onset PE compared to healthy pregnant controls (42).

Overall, 80.8% of the studies included in our review reported at least one of the following abnormalities in ANS function: parasympathetic withdrawal, sympathetic hyperactivity or reduced baroreflex sensitivity. ANS dysfunction was prevalent in 93.6% of the patients diagnosed with PE.

### Risk of Bias

The methodological quality of the included observational studies was tested using the Newcastle-Ottawa Scale (NOS) (53) which examines the risk of bias in observational studies by evaluating the selection of study groups, comparability of groups and ascertainment of either the exposure or outcome of interest. NOS consists of 8 items with 3 subscales, the total maximum score of these 3 subsets is 9. The outcomes of the quality assessment using the Newcastle–Ottawa Scale are presented in Table 2. A standard criterion for what constitutes a high-quality study has not yet been universally established. We considered a study that scored ≥7 a high-quality study. The mean value for the included studies is 7.6.

## Discussion

### Summary of Main Findings

#### Role of Dysautonomia in Preeclampsia

The major finding of our systematic review is that autonomic dysfunction seems to be a frequent sign in women with pre-eclamptic pregnancy which manifests as elevated sympathetic tone, reduced parasympathetic tone, and reduced baroreflex gain. These changes lead to a pattern of neural dysfunction which is dominated by impairment of cardiovascular autonomic function. The role of preeclampsia in the development of cardiovascular disturbances has been discussed to a rapidly growing extent. Women with a history of preeclampsia have a 2-fold higher risk of cardiovascular and cerebrovascular disease (54). Autonomic cardiovascular failure might contribute to this risk. An alternative (or complementary) explanation may be impairment of structural and functional integrity of the vasculature induced at the time of pre-eclamptic pregnancy as well as a higher cardiovascular risk burden in later life in women who had preeclampsia (55). These mechanisms appear to cause cumulative organ damage in pre-eclamptic women progressing even beyond the time of pregnancy. It was recently shown that previously pre-eclamptic women have greater cerebral structural changes than women who have normotensive pregnancies (56). This damage was most pronounced in the temporal lobe and increased with time, consistent with continued cumulative damage post pregnancy. Whether autonomic dysfunction contributes to brain changes beyond pregnancy remains uncertain. It is however interesting that brain changes in pre-eclamptic women appear to target the temporal lobe which harbors the insular center of autonomic cardiovascular control. It remains speculative if changes seen on autonomic function tests correlate with these structural changes.

Most research investigating the pathophysiology of preeclampsia showed less attention toward a possible role of the ANS.

Studies in healthy pregnancy compared to non-pregnant women showed that pregnancy itself, even when uncomplicated by preeclampsia, is characterized by an increase in sympathetic tone and a decrease in respiratory sinus arrhythmia together with. significantly elevated heart rate supports the existence of an underlying increase in sympathetic cardiac activity (35).

Studies in women diagnosed with PE indicate that, pre-eclamptic women showed higher cardiac output and heart rate which is regarded as a sign of increased sympathetic activity in addition to exaggerated peripheral vascular resistance and higher blood pressure which can be mediated, in part, by a substantial increase in sympathetic vasoconstrictor activity (52).

The role of sympathetic nervous activity SNA in the pathophysiology of PE has been extensively studied. A possible mechanism could be that early elevations in SNA encourage placental ischemia/reperfusion events and hypoxia-induced release of pro-hypertensive factors into the maternal circulation (8). It is also possible that placental ischemic factors reduce the vasoconstriction-buffering mechanisms in the blood vessel wall supporting the development of hypertension (57, 58).

However, the consistency of observations on cardiovascular autonomic dysfunction in pre-eclamptic women among studies included in our review indicates a potential pathophysiological role of dysautonomia in the course of PE which seems to exceed what can be explained by cumulative cardiovascular risk factors alone.

Furthermore, the relationship between fetal and maternal autonomic balance appears to play a role in the development of cardiovascular complications during or after pre-eclamptic pregnancy. Interestingly, the patterns of autonomic dysfunction seem to differ between pre-eclamptic women and their offspring. While maternal sympathetic overactivity modulates HRV by suppressing parasympathetic tone both in mild and severe cases of PE, fetal cardiac dysautonomia appears to be dominated by an increase in a sympathetic tone which leads to the suppression of fetal biophysical activity and the development of fetal distress in cases of severe PE (26).

Another approach to improve our understanding of autonomic dysfunction in pre-eclamptic women is to study the neuroendocrine axis. Normal pregnancy is associated with dramatic changes in hemodynamics and is accompanied by changing levels of various pressor hormones and vasoactive metabolites (59). In studies of pregnant women, biochemical biomarkers of sympathetic activity showed conflicting results which may be due to the fact that their levels are influenced by various factors such as activity of the neural efferent, release of the synaptic transmitter, reuptake mechanisms and regional blood flow (60), in addition to the fact that pregnancy itself affects the production and clearance of catecholamines (61). However, it is well-established that disturbed placentation and placental functioning in early pregnancy leads to inadequate spiral artery remodeling and thereby to chronic placental ischemia (62). Reactive oxygen species and cytokines released from the ischemic placenta as well as acute phase proteins trigger systemic oxidative stress and inflammatory response which then provokes the release of antiangiogenic factors. These factors inhibit angiogenesis and vasodilatation which result in endothelial dysfunction and increase arterial stiffness (63–67). Increased sympathetic activity was found in normotensive pregnant women, and it was even greater in women with gestational hypertension and preeclampsia at term (68). Since both endothelial function and arterial stiffness are in parts subject to autonomic neural control (predominantly sympathetic), it seems plausible that autonomic and antiangiogenic pathways are interlinked in the pathogenesis of preeclampsia and its cardiovascular complications in later life (69).

#### Predictive Role of Autonomic Function Testing

Studies of microneurography showed that MSNA levels are elevated in normotensive pregnancy compared to non-pregnant controls with significantly increased levels in PE patients but have no value in predicting PE in high-risk women (39). These findings are consistent with a study, which proved increased MSNA levels and impaired cardiac baroreceptor gain in patients with hypertensive pregnancy disorders (PIH and PE) and that MSNA levels returned to normal level after delivery (70). However, a long term follows up study showed that in previously pre-eclamptic women with treated hypertension sympathetic outflow is increased compared to normotensive control women despite similar ambulatory blood pressure values. Remarkably, this observation was made 40 years after the pre-eclamptic pregnancy indicating that long sympathetic changes in those women who have preeclampsia and continue to have hypertension post pregnancy (71). Although signs and symptoms of preeclampsia become apparent late in pregnancy, there is some evidence, that the observed increased sympathetic activity, may already be present before the clinical presentation of preeclampsia (72, 73).

Different methods were used for the clinical assessment of autonomic cardiovascular control in women diagnosed with PE. Non-invasive assessment of autonomic cardiovascular control was evaluated as a predictive tool to early identify women at increased risk of developing PE. They have the advantage of bearing minimal risk for the mother and the fetus and can be repeated during pregnancy. However, their results are limited by the fact that autonomic regulation of blood pressure can be disturbed at several levels between the hypothalamus and the periphery (59).

Overall, studies evaluating autonomic tests as a predictive tool showed inconsistent results That may be due to methodological factors and study design. Most studies are cross-sectional with a few numbers of longitudinal studies conducted before or early in pregnancy. Also, the performance of the different cardiovascular tests is not uniform and standardized besides the difference in blood pressure measurement methods (50). Recent studies have shown promising results using other tools such as circulating small non-coding RNA as a predictive tool for PE in the first trimester (74).

### Limitations

The present review is limited by the case-control design of the majority of the studies, while results from the prospective trials showed controversial results regarding the predictive value of different ANS testing for early detection of PE. A further limitation is the methodological heterogeneity present between studies, regarding the different definitions of preeclampsia, the nature of included PE population (late onset PE, early onset PE, severe and mild PE).

## Conclusions

Autonomic dysfunction is highly prevalent in pre-eclamptic women and might contribute to their increased cardiovascular and cerebrovascular risk. Tests of cardiovascular autonomic function such as orthostatic stress and cold pressor tests might be helpful to identify subjects at risk and monitor disease progression.

Biomarkers of sympathetic activity do not seem to be reliable tools to assess the sympathetic function in preeclampsia. MSNA is elevated in normal pregnancy which is further augmented in PE and also shows long term changes in those women who have had preeclampsia and continue to have hypertension after delivery. However, the technique seems to have low value in predicting the risk of PE in high-risk patients.

Dysautonomia in PE may be alleviated by an easy-to-learn technique, the heart rate variability biofeedback. It has been shown to improve both autonomic functioning and perinatal anxiety and depression (75, 76).

It is noteworthy that at this stage autonomic function testing is not able to differentiate between mild and severe PE. However, severe but not mild PE is accompanied by loss of the maternal-fetal hemodynamic coupling as seen with maternal and fetal RSA in women diagnosed with PE.

Further studies are needed to demonstrate the predictive value of ANS testing and their applicability as an ambulatory test alone or in combination with other biomarkers to predict the risk of PE early in pregnancy. Selection of recruited PE patients according to the onset and severity of disease might help further elucidate the underlying pathophysiology.

## Data Availability

All datasets generated for this study are included in the manuscript and/or the supplementary files.

## Author's Note

This work is part of a Masters thesis of the Master's Program in Clinical Research, Center for Clinical Research and Management Education, Division of Health Care Sciences, Dresden international University, Dresden, Germany.

## Author Contributions

DY drafted the first version of the manuscript. APe, MH, BI, APi, and TS have made substantial contributions by reviewing the manuscript for intellectual content, language and design. IB, DY, and TS have made substantial contributions to drafting the figures and tables displayed in this article. TS is the corresponding author.

### Conflict of Interest Statement

The authors declare that the research was conducted in the absence of any commercial or financial relationships that could be construed as a potential conflict of interest.
